# Deciphering the complexities of mucosal innate lymphocytes: guardians and potential therapeutic targets

**DOI:** 10.1038/s12276-023-01092-z

**Published:** 2023-09-11

**Authors:** Hye Young Kim, Ji Hyung Kim

**Affiliations:** 1 Laboratory of Mucosal Immunology in the Department of Biomedical Sciences, Seoul National University College of Medicine, Seoul, South Korea; 2 Institute of Allergy and Clinical Immunology, Seoul National University Medical Research Center, Seoul, South Korea; 3 Department of Biotechnology, College of Life Sciences and Biotechnology, Korea University, Seoul, South Korea

**Keywords:** Innate lymphoid cells, Mucosal immunology

The human body, an intricately woven tapestry of cells and systems, has long coexisted with a vast array of microbial companions. These trillions of microorganisms, collectively known as the microbiota, inhabit our mucosal surfaces and contribute to the orchestration of immune responses, ultimately shaping our overall health^1,2^. The symbiotic dance between the microbiota and the host’s immune system, particularly through the engagement of mucosal innate lymphocytes, has emerged as a captivating area of research with profound implications for immunotherapy^3–5^.

In this special issue entitled “Mucosal innate lymphocytes,” we delve into the captivating world of these immune sentinels and their intricate interactions with the microbiota. The human body’s mucosal surfaces, from the intestines to the lungs, serve as dynamic interfaces where the immune system and microbiota engage in constant communication. Over the years, an extensive body of research has underscored the crucial role of mucosal innate lymphocytes in maintaining immune homeostasis and bridging the gap between microbial signals and immune responses.

The exploration by Park et al. introduces the concept of microbiota-dependent regulation of costimulatory and coinhibitory pathways. The review intricately weaves together the threads of the microbiota-host interaction, revealing how these interactions intricately shape mucosal immune responses. The intriguing correlation between the efficacy of cancer immunotherapies and the gut microbiota sets the stage for potential breakthroughs in immunotherapy. (Park et al.: 10.1038/s12276-023-01075-0).

Diving deeper into the realm of innate lymphocytes, Yoo and Oh highlighted unconventional immune cells residing within the gut mucosal barrier. Innate lymphoid cells (ILCs) and unconventional T cells we identified as vital players that respond dynamically to bacterial cues, fostering a delicate balance between innate immunity and adaptive immunity. The role of these unconventional immune cells in communicating signals between the host immune system and the gut microbiota unveils a layer of complexity that underscores the symbiotic relationship. (Yoo et al.: 10.1038/s12276-023-01088-9).

Ryu et al. further unravel the versatile roles of ILCs at the mucosal barrier, providing insight into their involvement in both the pathogenesis and resolution of mucosal tissue diseases. This comprehensive review navigates the intricacies of ILC biology, underlining their potential as therapeutic targets for mucosal diseases. The journey from understanding their functions in health and disease to the exploration of immune checkpoints and therapeutic interventions adds depth to the canvas of mucosal immune regulation. (Ryu et al.: 10.1038/s12276-023-01022-z).

As the spotlight shifts to antigen presentation, Kim and colleagues shed light on CD1-mediated immune responses in mucosal tissues. This review describes the molecular mechanisms underlying lipid antigen presentation and the pivotal role of these responses in inflammation, autoimmune diseases, and infections. With the potential to unlock novel therapeutic approaches, understanding CD1-mediated antigen presentation could provide new strategies for manipulating immune responses in mucosal tissues. (Kim et al.: 10.1038/s12276-023-01053-6).

The complex interplay of immune responses is further amplified by the intricate roles of γδ T cells, as explored by Kang et al. These unconventional defenders wield a double-edged sword, guarding the mucosa against pathogens while sometimes exacerbating inflammation and contributing to autoimmune diseases. This review explores the balance between the protective and detrimental functions of these cells, highlighting the need for a deeper understanding to harness their therapeutic potential. (Kang et al.: 10.1038/s12276-023-00985-3).

From the depths of the lungs emerges the realm of invariant natural killer T (iNKT) cells, as elucidated by Jeong et al. These cells, characterized by their unique glycolipid antigen recognition, play multifaceted roles in maintaining immunological equilibrium. Their roles in lung inflammatory diseases, such as asthma and infections, provide a comprehensive overview of their contributions to health and disease, painting a holistic picture of iNKT cell biology. (Jeong et al.: 10.1038/s12276-023-01024-x).

The journey concludes with a focus on pulmonary group 2 innate lymphoid cells (ILC2s) and their role in asthma. Thio and Chang traverse the landscape of ILC2s, highlighting their complex roles in initiating airway inflammation and coordinating immune responses. Understanding the factors that modulate the function of these cells, from inflammatory mediators to environmental and metabolic influences, holds promise for unraveling the intricate pathways underlying asthma. (Thio et al.: 10.1038/s12276-023-01021-0).

Collectively, these articles chart a remarkable journey through the intricate interplay of mucosal innate lymphocytes, the microbiota, and immunotherapy. As researchers delve into these uncharted territories, they are unlocking the therapeutic potential embedded within these interactions, which stand as a promising beacon on the horizon of medical advancements. As we embrace the complexities, mysteries, and promises that this frontier offers, we venture forth into a realm where the microbiota, immune system, and innovative therapeutics converge to redefine the boundaries of human health and disease.
